# Bench-Scale Membrane Reactor for Methylcyclohexane Dehydrogenation Using Silica Membrane Module

**DOI:** 10.3390/membranes11050326

**Published:** 2021-04-29

**Authors:** Masahiro Seshimo, Hiromi Urai, Kazuaki Sasa, Hitoshi Nishino, Yuichiro Yamaguchi, Ryoichi Nishida, Shin-ichi Nakao

**Affiliations:** Inorganic Membranes Research Center, Research Institute of Innovative Technology for the Earth (RITE), Kyoto 619-0237, Japan; urai@rite.or.jp (H.U.); sasa@rite.or.jp (K.S.); nishino@osakagas.co.jp (H.N.); yamaguti@osakagas.co.jp (Y.Y.); nishida@osakagas.co.jp (R.N.); nakao@rite.or.jp (S.-i.N.)

**Keywords:** methylcyclohexane dehydrogenation, silica membrane, counter-diffusion chemical vapor deposition, membrane reactor module

## Abstract

Methylcyclohexane-toluene system is one of the most promising methods for hydrogen transport/storage. The methylcyclohexane dehydrogenation can be exceeded by the equilibrium conversion using membrane reactor. However, the modularization of the membrane reactor and manufacturing longer silica membranes than 100 mm are little developed. Herein, we have developed silica membrane with practical length by a counter-diffusion chemical vapor deposition method, and membrane reactor module bundled multiple silica membranes. The developed 500 mm-length silica membrane had high hydrogen permselective performance (H_2_ permeance > 1 × 10^−6^ mol m^−2^ s^−1^ Pa^−1^, H_2_/SF_6_ selectivity > 10,000). In addition, we successfully demonstrated effective methylcyclohexane dehydrogenation using a flange-type membrane reactor module, which was installed with 6 silica membranes. The results indicated that conversion of methylcyclohexane was around 85% at 573 K, whereas the equilibrium conversion was 42%.

## 1. Introduction

In order to create a hydrogen energy–based society, transport and storage of hydrogen in one of the most important issues. Recently, the development of liquid hydrogen, ammonia, and organic chemical hydrides methods for hydrogen transport/storage have progressed in the world. Above all, organic chemical hydride methods such as the methylcyclohexane (MCH)–toluene system can be expected as the most practical ones, because they are liquid at normal temperature and pressure, and the existing infrastructure can be utilized.

The dehydrogenation of MCH is the equilibrium reaction. Its conversion ratio can exceed the equilibrium by removing hydrogen from the reaction system using the membrane reactor with hydrogen permselective membrane. Palladium-based or silica membranes which show hydrogen selective permeation performance are developed for MCH dehydrogenation membrane reactors. Palladium-based membranes are mainly prepared by electroless plating [1,2] and electroplating [3,4]. Because the reaction temperature of MCH dehydrogenation is operated at 573–673 K, Pd–Ag alloy membrane was usually applied to the membrane reactor owing to the suppression of hydrogen embrittlement [5,6]. On the other hand, the palladium-based membranes have the disadvantage that membrane costs are high. Amorphous silica membranes are prepared by sol–gel [7,8,9] or chemical vapor deposition [10,11,12] method. Hydrogen permeation performance of membranes greatly affects the reaction efficiency when we use membrane reactors; therefore, higher hydrogen permeance is desirable for the practical application of membrane reactors for the MCH dehydrogenation. It is possible to obtain high hydrogen permeance by loosely tuning the pore diameter. For both preparation methods, pore size of the silica membrane can be controlled relatively easily by changing silica precursor [13,14,15]; therefore, these membranes can be applied to the membrane reactor for MCH dehydrogenation [16,17]. Durability of silica membranes is also important. Durability of the membrane reactor using silica membranes was already evaluated by Akamatsu et al. [18]. These results show promising possibility of membrane reactors; however, they are all results at laboratory scale level.

From the view point of practical use of membrane reactors, developing silica membranes with practical length, approximately 500 mm, and modular structures of the membranes, are very important. However, there are very few researchers who have attempted to develop such silica membranes or module structures. Since MCH dehydrogenation is an endothermic reaction, a method and module structure for efficiently supplying the reaction heat is important.

In this study, to advance social implementation, we have developed long-scale DMDPS–derived silica membrane having high H_2_ permselective performance prepared by counter-diffusion chemical vapor deposition method, and module of six silica membranes. We also evaluated the performance of bench–scale membrane reactor including multiple silica membranes for methylcyclohexane dehydrogenation. In addition, improvement of heat transfer to catalyst layer was also investigated.

## 2. Materials and Methods

### 2.1. Preparation and Permeation Perfomance Measurement of DMDPS-Derived Silica Membranes

Longer silica membranes having practical length were prepared and evaluated. We employed counter–diffusion chemical vapor deposition method for silica membranes preparation up to 500 mm long.

**Figure 1:**
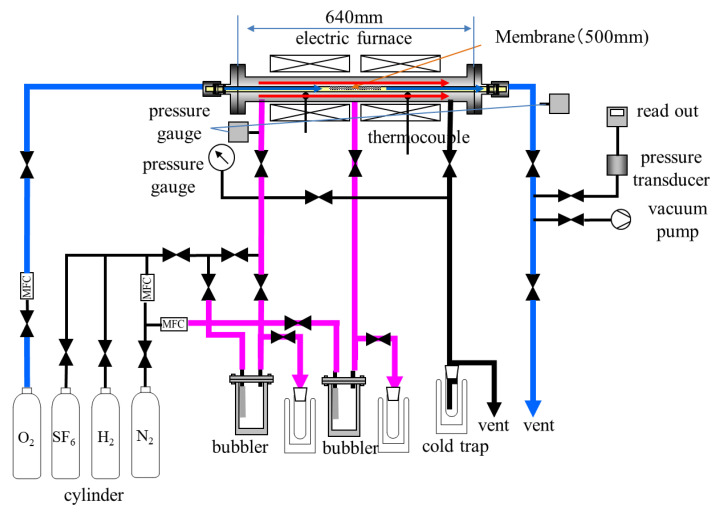
Schematic diagram of experimental apparatus for 500 mm–length silica membrane preparation and performance measurement.

Figure 1 shows a schematic diagram of apparatus for longer membrane preparation and their permeation performance measurement. The silica membrane derived from dimethoxydiphenylsilane was formed on a porous alumina tube. An α–alumina tubular support was purchased from Nikkato corporation, Osaka, Japan, and a γ–alumina layer was coated on the support by sol–gel method. Saturated dimehoxydiphenylsilane (DMDPS, Shin-Estu Chemical Co., Ltd., Tokyo, Japan) vapor was fed to the exterior of the γ–alumina coated support with N_2_, as carrier gas, and the vapor concentration was regulated at 0.1 mol/m^3^ by controlling the temperature of a bubbler. O_2_, at rate of 250 mL/min, was introduced to the interior of the support. An amorphous silica layer derived from DMDPS was deposited into the pores and surface of the support. Reaction temperature and time for CVD were 873 K and 60 min, respectively. Permeation performance of obtained silica membrane was evaluated at 573, 473, 373, and 298 K, using single component H_2_, N_2_ and SF_6_ gases. Flow rate of H_2_ and N_2_ in permeation side was measured by soap film flow meter, and permeances were calculated from these rates. Determination of SF_6_ permeance was performed with a pressure difference method.

### 2.2. Bench–Scale Membrane Reactor Test for Methylcyclohexane Dehydrogenation

**Figure 2:**
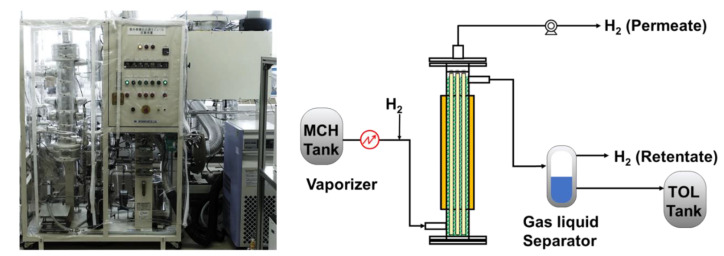
Picture and schematic diagram of small-scale membrane reactor for methylcyclohexane dehydrogenation.

Bench–scale membrane reactor tests using a module in which multiple silica membranes are bundled were conducted. The experimental apparatus for methylcyclohexane dehydrogenation membrane reactor is shown in Figure 2. Pt catalysts were placed outside of the membranes, and the reactor module included silica membranes was heated using hot oil to reaction temperature. Before the reaction tests, the catalysts were reduced under hydrogen atmosphere. Methylcyclohexane, which is a reaction raw material, was fed to the exterior of the membranes through a vaporizer, and the dehydrogenation reaction was conducted at 573 K. The pressure of the reaction and permeation sides were maintained at 0.4 MPaA and 0.1 MPaA, respectively. To calculate methylcyclohexane conversion, the concentrations of methylcyclohexane, toluene, and hydrogen in the retentate and permeate side were measured by a gas chromatograph (7820A, Agilent Technologies Japan, Ltd., Tokyo, Japan). During membrane reactor tests, no sweep gas was flowing to the permeation side. In addition, the overall heat transfer coefficient was calculated using temperature of the inlet and outlet.

## 3. Results and Discussion

### 3.1. Permeation Performance of 500 mm-length DMDPS-Derived Silica Membrane

**Figure 3:**
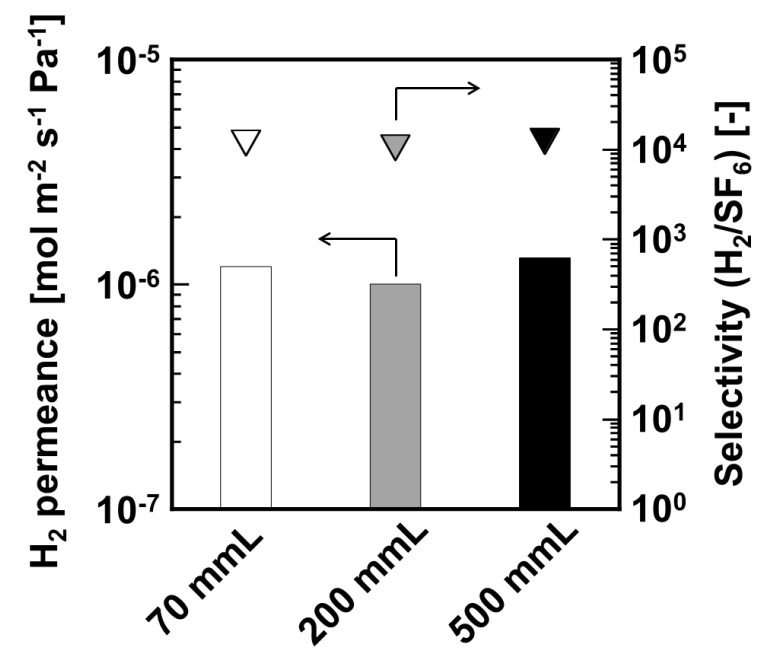
Hydrogen permeation performance of DMDPS-derived silica membrane having different effective membrane lengths at 573 K.

Comparative permselective performances of DMDPS–derived silica membranes formed on different effective membrane lengths as 70, 200, and 500 mm–length are shown in Figure 3. Permeation measurement of these membranes was conducted at 573 K. H_2_ permeances of 70, 200, and 500 mm-length membranes were 1.20 × 10^−6^, 1.00 × 10^−6^, and 1.30 × 10^−6^ mol m^−2^ s^−1^ Pa^−1^, respectively. H_2_/SF_6_ selectivity of these membranes was 12,000, 10,000, and 12,000, respectively. Here, the H_2_/SF_6_ selectivity is used as an indicator for H_2_/toluene selectivity, because the kinetic diameter of toluene is similar to that of SF_6_. All membranes showed approximately the same H_2_ permselective performance despite different membrane lengths. From these results, we successfully formed long–scale silica membrane.

**Figure 4:**
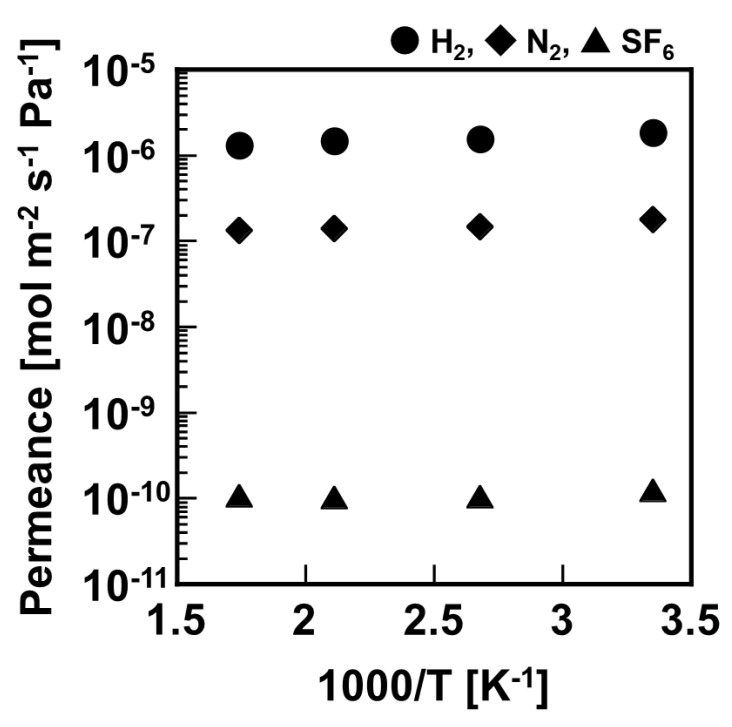
Temperature dependence of permeation performance through 500 mm-length silica membrane derived from DMDPS at 573–298 K.

Figure 4 shows temperature dependence of H_2_, N_2_, and SF_6_ permeation through 500 mm–length silica membrane in the temperature range of 573–298 K. Permeances of H_2_, N_2_, and SF_6_ showed an approximately constant value regardless of this temperature range. At 573 K, the permeances of H_2_, N_2_, and SF_6_ were 1.30 × 10^−6^ mol m^−2^ s^−1^ Pa^−1^, 1.31 × 10^−7^ mol m^−2^ s^−1^ Pa^−1^, and 1.07 × 10^−10^ mol m^−2^ s^−1^ Pa^−1^, respectively. The selectivity of H_2_/N_2_ was 10, and that of H_2_/SF_6_ was 12,000 at 573 K. The temperature dependence and permselectivity of this 500 mm-length silica membrane showed approximately similar performance as in a previous report of developed 100 mm-length DMDPS–derived silica membrane [19,20].

### 3.2. Bench–Scale Membrane Reactor including Multiple Silica Membranes for Methylcyclohexane Dehydrogenation Reaction

**Figure 5:**
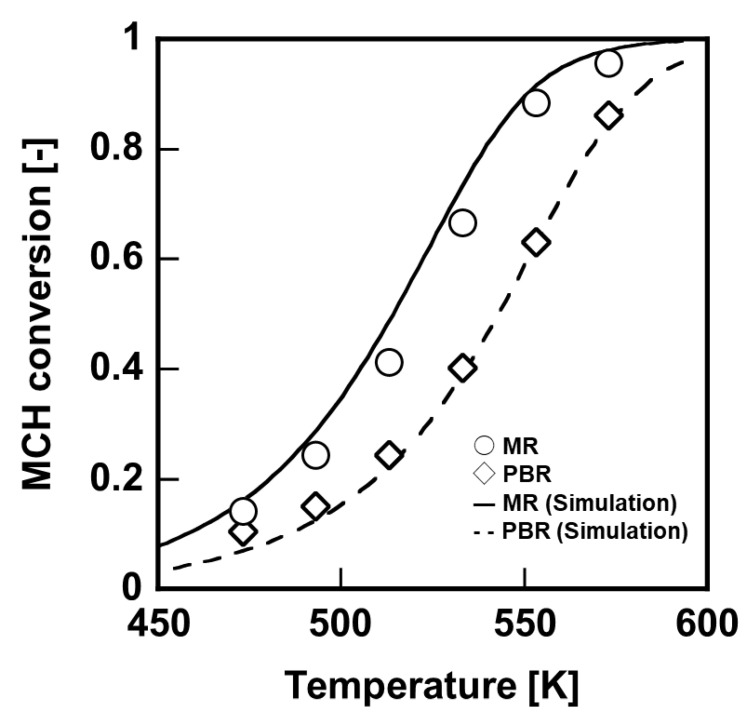
Temperature dependence of the MCH conversion to compare between packed–bed reactor (PBR) and membrane reactor (MR) which was included in a single silica membrane.

First, dehydrogenation membrane reactor, which was included a single silica membrane having 70 mm–length, was evaluated under 0.2 MPaA to compare between performance of packed-bed reactor and membrane reactor, as shown in Figure 5. The MCH conversion ratio with membrane reactor was much larger than that with packed-bed reactor in all temperature range, owing to the hydrogen extraction by the silica membrane. In order to obtain the MCH conversion of 90%, the reaction temperature required was 583 K in the packed-bed reactor; however, it was experimentally shown that the reaction temperature could be reduced to 553 K by using the membrane reactor.

A novel membrane module in which 6 silica membranes having 200 mm–length were directly bonded with glass sealant to a metal flat plate was developed as one of the modularization technologies with mass production possibility, as shown in Table 1. Permselective performances of 6 silica membranes derived from DMDPS at 573 K are shown in Table 1. These membranes were prepared using the same conditions. All the membranes had over 1 × 10^−6^ mol m^−2^ s^−1^ Pa^−1^ of hydrogen permeance. On the other hand, the H_2_/SF_6_ selectivity of 6 silica membranes was slightly different. The SF_6_ permeance showed an extremely low value (around 1 × 10^−10^ mol m^−2^ s^−1^ Pa^−1^), which is closed to the measurement limit; therefore, it was considered that there are few significant differences in the H_2_/SF_6_ selectivity between 6 silica membranes. Table 2 shows the permselective performances of our previous silica membranes for MCH dehydrogenation. Compared with previous works, H_2_ permeance was around 2 times higher because of changing to tubular alumina support having relatively high gas permeation performance. These membranes were applied to the flange–type membrane module. In addition, this membrane module structure had confirmed hydrogen tightness under 573 K, 0.5 MPa.

**Figure 6:**
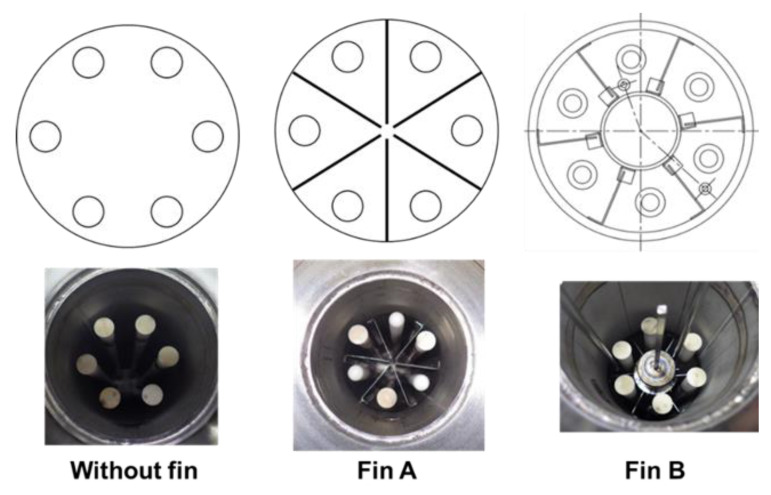
Structures of heat conductive fins to improve heat transfer from the heat medium to the catalyst layer.

The MCH dehydrogenation is an endothermic reaction. When the reaction is conducted with a membrane reactor, the conversion ratio is decreased owing to reaction temperature reduction. Therefore, we evaluated the effect of heat conductive fins to improve the temperature uniformity of catalyst layer. We tried two different structures of heat conductive fins, as shown in Figure 6.

**Figure 7:**
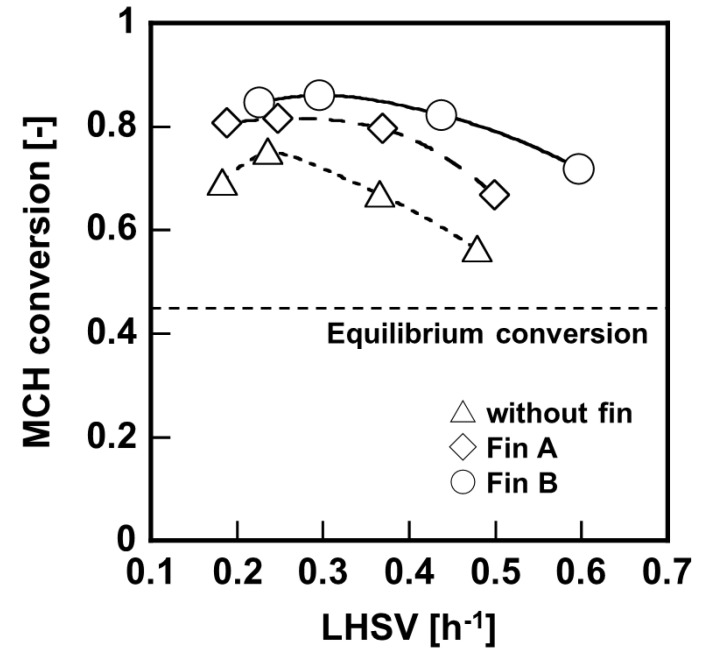
Comparison of MCH conversion using membrane reactor with different structure of heat conductive fins.

**Figure 8:**
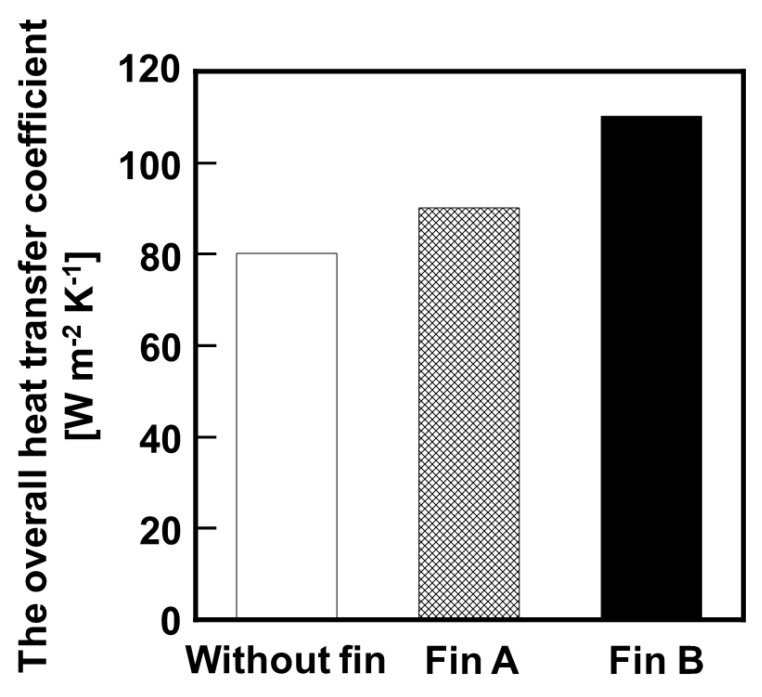
Comparison of the overall heat transfer coefficient using different structure of heat conductive fins.

Results of bench–scale membrane reactor tests using different structure of heat conductive fins are shown in Figure 7, and the overall heat transfer coefficient calculated from temperature of the inlet and outlet is shown in Figure 8. In both cases, the MCH conversion ratio exceeded the equilibrium conversion; however the reactors with heat conductive fins were performed relatively high MCH conversion ratio to compared with that without heat conductive fin. In addition, the reactor using Fin B showed higher conversion than that using Fin A under relatively high LHSV condition. The overall heat transfer coefficients of the reactor without fin, with Fin A, and with Fin B were 80, 90, and 110 W m^−2^ K^−1^, respectively. These results suggested that the installation of heat conductive fins effectively transferred heat to the catalyst layer, and promoted the MCH dehydrogenation reaction.

## 4. Conclusions

We successfully demonstrated the preparation of practical long silica membranes with high hydrogen permselective performance, and effective MCH dehydrogenation using a novel membrane reactor module bundled with 6 silica membranes. Developed 500 mm-length silica membrane had over 1 × 10^−6^ mol m^−2^ s^−1^ Pa^−1^ of hydrogen permeance, and over 10,000 of H_2_/SF_6_ selectivity. The membrane reactor module was evaluated at 573 K, 0.4 MPaA. The MCH conversion using membrane reactor exceeded the equilibrium conversion owing to hydrogen extraction effect. In addition, we clarified that improvement of heat transfer to catalyst layer was an important issue by using the membrane reactor module improved overall heat transfer coefficient, because MCH dehydrogenation is an endothermic reaction.

## Figures and Tables

**Table 1:** Hydrogen permselective performances of silica membranes for membrane reactor module.

No.	H_2_ Permeance [mol m^−2^ s^−1^ Pa^−1^]	Selectivity (H_2_/SF_6_) [–]
1	2.84 × 10^−6^	21,300
2	2.00 × 10^−6^	15,100
3	1.94 × 10^−6^	13,100
4	2.09 × 10^−6^	9930
5	2.04 × 10^−6^	39,300
6	1.87 × 10^−6^	10,300
Picture of a novel module using No.1 ~ 6 membranes	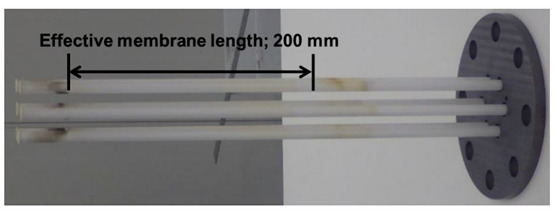	

**Table 2:** Hydrogen permselective performances of our previous silica membranes for MCH dehydrogenation.

Ref.	H_2_ Permeance [mol m^−2^ s^−1^ Pa^−1^]	Selectivity (H_2_/SF_6_) [–]
[18]	1.2 × 10^−6^	9300
[19]	1.17 × 10^−6^	4980
[20]	1.09 × 10^−6^	19,300

## Data Availability

Not applicable.
